# Adult Intra-Thoracic Kidney: A Case Report of Bochdalek Hernia

**DOI:** 10.1155/2010/975168

**Published:** 2010-08-30

**Authors:** Valeria Fiaschetti, Luca Velari, Eleonora Gaspari, Roberta Mastrangeli, Giovanni Simonetti

**Affiliations:** Department of Diagnostic Imaging, Molecular Imaging, Interventional Radiology and Radiation Therapy, University Hospital, “Tor Vergata”, 81 Oxford street, 00133 Rome, Italy

## Abstract

*Introduction*. Bochdalek hernia is a congenital posterior lateral diaphragmatic defect that allows abdominal viscera to herniate into the thorax. Intrathoracic kidney is a very rare finding representing less than 5% of all renal ectopias with the least frequency of all renal ectopias. *Case Presentation*. We report a case of a 62-year-old man who had a left thoracic kidney associated with left Bochdalek hernia. Abdominal X-ray and chest X-ray revealed dilated loops of the colon above left hemidiaphragm. Abdominal ultrasound (US) showed the right kidney with many fluid and esophytic cysts; left kidney was unfeasible to study because of the impossibility to find it. Computed Tomography (CT) basal scan demonstrated a left-sided Bochdalek hernia with dilatated colon loops and the left kidney within the pleural space. Magnetic Resonance (MR) confirmed a defect in left hemidiaphragm with herniation of left kidney, omento, spleen and colon flexure, and intrarotation with posterior hilum on sagittal plane. *Conclusion*. The association of a Bochdalek hernia and an intrathoracic renal ectopia is very rare, that pose many diagnostic and management dilemmas for clinicians. Our patient has been visualized by CT and MR imaging. A high index of suspicion can result in early diagnosis and prompt intervention with reduced morbidity and mortality.

## 1. Introduction

Bochdalek hernia is a congenital posterior lateral diaphragmatic defect that allows abdominal viscera to herniate into the thorax [1].

It is the most common type of congenital diaphragmatic hernias and occur in approximately 1 in 2,200–12,500 live births; they are seen with much greater frequency on the left hemithorax and associated to a normal diaphragm [2, 3].

Intra-thoracic kidney is a very rare finding representing less than 5% of all renal ectopias with the least frequency of all renal ectopias [4–6]; most are found in males and are asymptomatic. The incidence of intra-thoracic renal ectopia as a result of congenital diaphragmatic hernia was reported to be less than 0.25% [4]. 

We report a case of a man who had a left thoracic kidney associated with left Bochdalek hernia.

## 2. Case Report

A 62-year-old man came to our centre to make a chest X-ray and abdominal X-ray. He referred to cough from 1 month, abdominal pain particularly post-prandial, and difficult to urinary.

**Figure 1:**
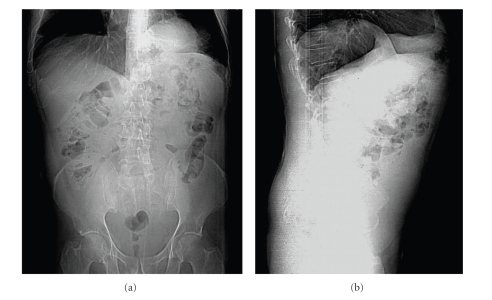
Abdominal X-ray showed a dilated loops of the colon above left hemidiaphragm.

Abdominal X-ray and chest X-ray revealed a dilated loops of the colon above left hemidiaphragm (Figure 1). He did not suffer respiratory distress or recurrent pleural effusion.

**Figure 2:**
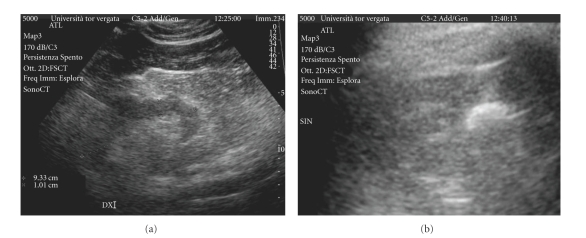
Ultrasonography imaging: normal right kidney (a) and absence of left kidney in the renal space (b).

The patient underwent also renal and bladder ultrasound (ATL HDI 5000); the right kidney presented many fluid cysts, a few with esophytic growth. Left kidney was unfeasible to study because of the impossibility to find it. Bladder's wall was thickened (Figure 2).

Radiologist decided to perform Computed Tomography (CT) study in order to evaluate left kidney and bladder.

Computed Tomography basal scan was performed because of serum creatinine levels of 2,6 mg/dl and azotemia value of 89 mg/dl.

**Figure 3:**
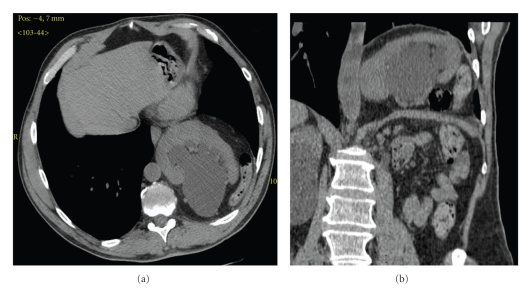
Basal CT (computed tomography) imaging. (a) Axial scan illustrates the left renal ectopia with renal junction expanded. (b) multiplanar reconstruction (MPR) on coronal plane confirm Bochdalech hernia.

Computed tomography showed a left sided Bochdalek hernia with dilatated colon loops and the left kidney within the pleural space. The intra-thoracic kidney presented a hilum in posterior position and an elongated and expanded ureteropelvic junction and the remaining portion of ureter. The contralateral kidney presented multiple esophytics cysts, with regular urinary tract (Figure 3).

To make a functional study of patient, a high field (3T) Magnetic Resonance (Intera, Philips Medical Systems, Best, Netherlands) was performed. After a survey scan and reference scan, an axial T1 turbo spin echo (TSE), axial STIR, and T2 weighted breath hold were used both in axial, coronal, and sagittal plane with a 2 mm thickness partition without a gap.

A bolus injection of gadolinium (Gd) Gadoteridol (ProHance) at the standard single dose of 0,1 mmol/kg of body weight was administered at the rate of 2,5 mL/sec, using an automatic injector to make urographic study.

Postprocessing included multiplanar reconstructions (MPRs). Magnetic Resonance Imaging (MRI) shows a defect in left hemidiaphragm with erniation of left kidney, omento, spleen and colon flexure. 

**Figure 4:**
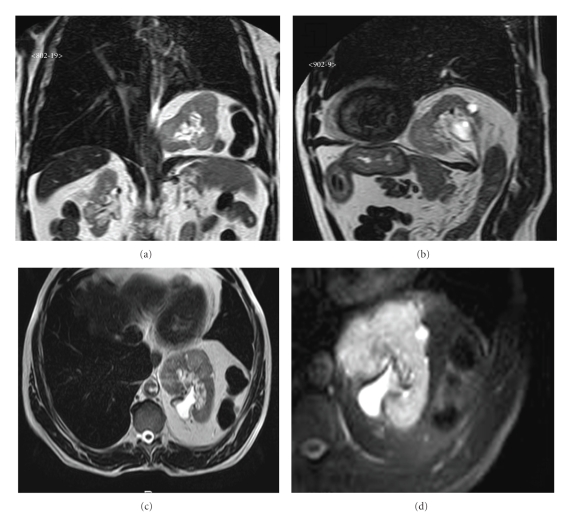
Magnetic resonance imaging (MRI). (a) T2 sequence on coronal view. (b) T2 sequence on sagittal plane. (c) T2-weighted image on axial plane. (d) Particular of left kidney on T2 axial view with fat suppression.

**Figure 5:**
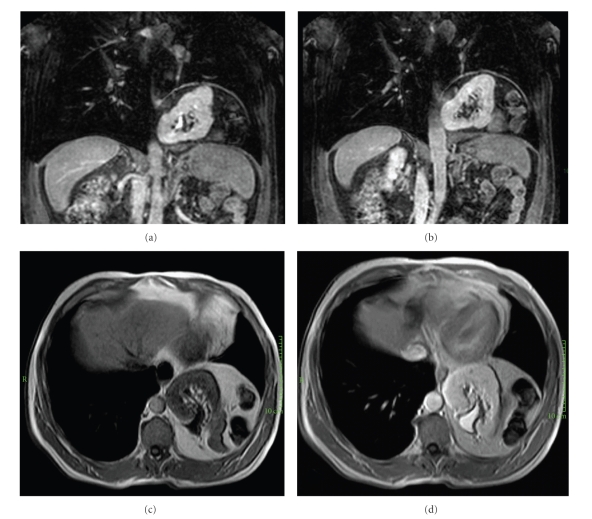
Magnetic resonance imaging (MRI). (a and b) THRIVE sequences with bolus of contrast medium injection; (c) axial T1-weighted sequence; (d) T1 post-Gd DTPA.

MRI confirmed left kidney intra-rotation with posterior hilum on sagittal plane. Contrast-enhanced sequences demonstrated normal renal arteries; a perfusion delay compared to right kidney was observed due to traction phenomena of vascular pedicle (Figures 4 and 5).

Patient was invited to urologic and nephrologic examination.

## 3. Discussion

Bochdalek's hernia (posterolateral defect, pleuroperitoneal hernia), firstly described by Bochdalek in 1848 [7], is a congenital posterior lateral diaphragmatic defect that allows abdominal viscera to herniate into the thorax, resulting from failed closure at 8 weeks of gestation of the pleuroperitoneal ducts, primitive communications between the pleural and abdominal cavities [1, 3]. It is more common in infants (90%) with an incidence of 1/2500 live births; however, the literature on Bochdalek hernia in adulthood is rather limited, with approximately 100 cases reported [2, 8–14] even if asymptomatic prevalence in the general population may be as high as 0.17% [10, 15]. It occurs most frequently on the left side with approximately 80% being left-sided and 20% right-sided [16]. This is presumably due to the pleuroperitoneal canal closes earlier on the right side [17], or to narrowing of the right pleuroperitoneal canal by the caudate lobe of the liver [18].

Bilateral Bochdalek's hernias are rare [16, 17]. These hernias are usually congenital and may cause severe life-threatening respiratory distress in the first hours or days of life. Herniated organs are frequently the omentum, bowel, spleen, stomach, kidney, and pancreas on the left, and part of the liver on the right. Because of the pulmonary hypoplasia due to the compression of the lungs by the adjacent hernia, these patients are frequently symptomatic at birth.

Although this condition usually presents in the neonatal period with severe respiratory distress, a few cases being asymptomatic until adult life have also been reported in literature and are usually associated with a better outcome [19–21].

In childhood, they are often misdiagnosed as pleuritis, pulmonary tuberculosis, or pneumothorax, and this can result in significant morbidity.

In adults, like infants, most occur on the left side (85%), usually causing gastrointestinal symptoms. In contrast to the acute presentation by infants with these hernias, most adults present with more chronic abdominal symptoms [22], such as recurrent pain, vomiting, and postprandial fullness [23]. Chronic dyspnea, pleural effusion, and chest pain are the most common chest symptoms and signs that are present in this condition [8].

Diagnosis requires a high suspicious index and needs to be confirmed with image studies. In adults, Bochdalek's hernias are diagnosed incidentally but most cases become surgical emergencies when an abdominal organ is strangled [3]. While urgent surgery is frequently needed for the treatment of the symptomatic Bochdalek hernia, the surgical treatment of asymptomatic Bochdalek hernias may be performed days to years later according to the patient's status. Larger hernias should be operated because of potential complications.

Renal ectopia describes a kidney that is not located in its usual position. Ectopic kidneys are thought to occur in approximately 1 in 1,000 births, but only about 1 in 10 of these are ever diagnosed [6]. 

Some of these are discovered incidentally, such as when a child or adult is having surgery or an X-ray for a medical condition unrelated to the renal ectopia. 

The complex embryological development of the kidneys can lead to renal anomalies, such as renal ectopia. Most ectopic kidneys are found in the lower lumbar or pelvic region secondary to failure to ascend during fetal life [24].

With a prevalence of less than 0.01%, intra-thoracic kidneys represent less than 5% of all renal ectopias with the least frequency of all renal ectopias [4–6].

Wolfromm [25] reported the first case of clinically diagnosed intra-thoracic kidney in 1940. In 1988, S. M. Donat and P. E. Donat [4] reviewed cases reported in the literature between 1922 and 1986, and found the abnormality to occur more commonly on the left (62%) than on the right side (36%); 2% of the patients had bilateral intra-thoracic kidney. In addition, this anomaly is observed with higher frequency in males (63%) than in females (37%) [26]. 

Pfister-Goedeke and Burnier [27] classified the thoracic kidneys into 4 groups: thoracic renal ectopia with closed diaphragm, eventration of the diaphragm, diaphragmatic hernia (congenital diaphragmatic defects or acquired hernia such as Bochdalek hernia), and traumatic rupture of the diaphragm with renal ectopia.

The incidence of intra-thoracic kidney with Bochdalek hernia is reported to be less than 0.25% [4], and the relationship between them remains uncertain. The embryological origin is debatable: various authors have proposed that there exists either an abnormality in the pleuroperitoneal membrane fusion or an abnormality in the high migration of the kidney due to delayed mesonephric involution [28].

Intra-thoracic kidney associated with Bochdalek hernia differs from other intra-thoracic renal ectopias as it tends to be mobile and easily reduced from the thorax to the abdominal cavity with other organs. [26] Commensurate herniation of abdominal viscera is common.

In all cases, the kidney is located within the thoracic cavity and not in the pleural space; the renal vasculature and ureter on the affected side typically exit the pleural cavity through the foramen of Bochdalek and are usually significantly longer than those in the normally positioned kidney [29]. Most intra-thoracic kidneys remain asymptomatic and have a benign course [30]. 

Anatomically, the features of intra-thoracic kidney are rotational anomalies such as the hilus facing posteriorly, long ureter, high origin of the renal vessels, and occasionally medial deviation of the lower pole of the kidney [26, 31, 32]. 

In spite of these abnormalities, it is usually fully functional and does not exhibit dysplasia, contralateral hypertrophy, or obstruction of the lower urinary tract [4, 25–27, 33–35]. 

Treatment for the ectopic kidney is only necessary if obstruction or vesicoureteral reflux (VUR) is present. There is an increased incidence of ureteropelvic junction obstruction, VUR, and multicystic renal dysplasia in ectopic kidney [6, 29]. 

If the kidney is not severely damaged by the time the abnormality is discovered, the obstruction can be relieved or the VUR corrected with an operation. However, if the kidney is badly scarred and not working well, removing it may be the best choice [6, 29].

Our patient had an elongated ureter, medially deviated lower pole, and rotational abnormality in which the hilum was posterior. The left intra-thoracic kidney and the left Bochdalek hernia in our patient has been visualized by CT and MR imaging.

Intra-thoracic kidneys are rare clinical entities that pose many diagnostic and management dilemmas for clinicians. The association of a Bochdalek hernia and an intra-thoracic renal ectopia is very rare. It is emphasized that this condition should be considered in the differential diagnosis of a lower intra-thoracic mass. A high index of suspicion can result in early diagnosis and prompt intervention with reduced morbidity and mortality.
